# A fragmented metazoan organellar genome: the two mitochondrial chromosomes of *Hydra magnipapillata*

**DOI:** 10.1186/1471-2164-9-350

**Published:** 2008-07-26

**Authors:** Oliver Voigt, Dirk Erpenbeck, Gert Wörheide

**Affiliations:** 1 Courant Research Center Geobiology, Georg-August-Universität Göttingen, Goldschmidtstr. 3, 37077 Göttingen, Germany

## Abstract

**Background:**

Animal mitochondrial (mt) genomes are characteristically circular molecules of ~16–20 kb. Medusozoa (Cnidaria excluding Anthozoa) are exceptional in that their mt genomes are linear and sometimes subdivided into two to presumably four different molecules. In the genus *Hydra*, the mt genome comprises one or two mt chromosomes. Here, we present the whole mt genome sequence from the hydrozoan *Hydra magnipapillata*, comprising the first sequence of a fragmented metazoan mt genome encoded on two linear mt chromosomes (mt1 and mt2).

**Results:**

The *H. magnipapillata *mt chromosomes contain the typical metazoan set of 13 genes for respiratory proteins, the two rRNA genes and two tRNA genes. All genes are unidirectionally oriented on mt1 and mt2, and several genes overlap. The gene arrangement suggests that the two mt chromosomes originated from one linear molecule that separated between *nd5 *and *rns*. Strong correlations between the AT content of rRNA genes (*rns *and *rnl*) and the AT content of protein-coding genes among 24 cnidarian genomes imply that base composition is mainly determined by mt genome-wide constraints. We show that identical inverted terminal repeats (ITR) occur on both chromosomes; these ITR contain a partial copy or part of the 3' end of *cox1 *(54 bp). Additionally, both mt chromosomes possess identical oriented sequences (IOS) at the 5' and 3' ends (5' and 3' IOS) adjacent to the ITR. The 5' IOS contains *trnM *and non-coding sequences (119 bp), whereas the 3' IOS comprises a larger part (mt2) with a larger partial copy of *cox1 *(243 bp).

**Conclusion:**

ITR are also documented in the two other available medusozoan mt genomes (*Aurelia aurita *and *Hydra oligactis*). In *H. magnipapillata*, the arrangement of ITR and 5' IOS and 3' IOS suggest that these regions are crucial for mt DNA replication and/or transcription initiation. An analogous organization occurs in a highly fragmented ichthyosporean mt genome. With our data, we can reject a model of mt replication that has previously been proposed for *Hydra*. This raises new questions regarding replication mechanisms probably employed by all medusozoans, and also has general implications for the expected organization of fragmented linear mt chromosomes of other taxa.

## Background

Mitochondria were most likely acquired by the common ancestor of eukaryotes [1-3]. Presumably, these organelles originated from incorporated α-proteobacteria and still carry their own, reduced genome [1]. Mitochondrial (mt) genomes show very diverse organizations and are of a very broad range of sizes [3-5]. In comparison to many protists and plants, metazoans possess an even more reduced set of mt genes and fewer non-coding regions [6]. Typical metazoan mt genomes are circular DNA molecules of 16–20 kb [6,7]. Remarkable exceptions are the linear mt genomes of medusozoan cnidarians (Cnidaria excluding Anthozoa). Linear mt genomes have not been found in other metazoan taxa, but in various other eukaryotes (e.g., [8-12]). The linear structure of cnidarian mt genomes is known from the work of Warrior [13], who separated the DNA of isolated mitochondria via electrophoresis, as well as from Bridge et al. [14] and Ender and Schierwater [15], who applied *rnl*-probes to electrophoretically separated DNA extracts. Most of the medusozoan mt genomes from these studies were encoded on one ~16 kb molecule, which has been verified by the first two sequences of such linear metazoan genomes (*Aurelia aurita *(Scyphozoa) [16], and *Hydra oligactis *(Hydrozoa) [17]). However, in some *Hydra *species, and apparently in some Cubozoa, the mt DNA is divided onto at least two different molecules [13-15]. In the genus *Hydra*, mt genomes are organized on one ~16 kb molecule or two ~8 kb molecules [13,14], making this genus an excellent candidate in which to examine changes due to fragmentation of mt genomes from one to two chromosomes. The *Hydra oligactis *mt genome, a 16.3 kb linear DNA molecule, was published recently [17]. Pont-Kingdon et al. [18] sequenced a terminal section (3,232 bp) of one of the two mt chromosomes from *Hydra vulgaris *(as *Hydra attenuata*), and previous hybridization experiments have shown that in this species all four termini possess a 150–200 bp identical sequence with unknown orientation to one another [13]. By providing the complete mt genome sequence of *Hydra magnipapillata*, encoded on two mt chromosomes, we now present in detail the organization of such a fragmented linear mt genome from early diverging Metazoa.

## Methods

**Figure 1:**
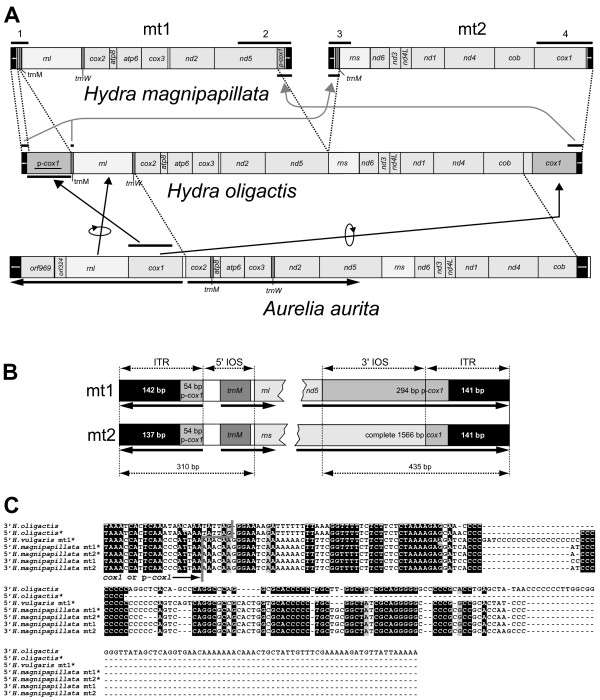
**Organization of the *H. magnipapillata *mt chromosomes (mt1 and mt2)**. **A: **In comparison to the linear mt genome of *H. oligactis *(Hydrozoa) and *Aurelia aurita *(Scyphozoa), drawn to scale. Arrows indicate orientation of genes in *Aurelia*. Numbered black bars above *H. magnipapillata *mt chromosomes correspond to the PCR fragments amplified from *Hydra *sp. (Additional file 3). Arrows in grey indicate the proposed duplications of terminal sequences in the mt chromosome separation process. **B: **Organization at the 5' and 3' ends of mt1 and mt2 in *H. magnipapillata*. Arrows in the inverted terminal repeats (ITR) are drawn according to the orientation of the *cox1 *fragment. **C: **Alignment of the ends of the ITR from *H. oligactis*, *H. vulgaris *(mt1) and *H. magnipapillata *(mt1 and mt2). * = sequence displayed as reverse complement.

We assembled the two mt chromosomes by using publicly available sequences from the *Hydra magnipapillata *whole-genome shotgun sequencing project by conducting BLAST searches [19] of several mt protein-coding genes against the traces of *H. magnipapillata *(available via GenBank [20]). Hits were used to initiate local genome assembly in a bioinformatical pipeline (applying the cap3 assembler [21]) to obtain the two mt chromosome sequences. The chromosomes will be referred to as mt1 (containing the *rnl *gene; available at [EMBL: BN001179]) and mt2 (containing the *rns *gene; available at [EMBL: BN001180]) (see also Additional file 1 for the list of trace IDs). mt1 and mt2 of *Hydra *share almost identical but inverted sequences of 191–196 bp at each 5' and 3' end (inverted terminal repeats (ITR), Figs. 1A, B, see below). Warrior [13] previously reported that about 200 bp were identical (with unknown orientation to one another) at the ends of the mt chromosomes of *Hydra vulgaris*, and the 5' end of one chromosome (corresponding to mt1) of this species was sequenced by Pont-Kingdon et al. [18]. By comparing our sequences with the experimentally verified 5' end of mt1 from *H. vulgaris *[18], we were able to infer the ends of the *H. magnipapillata *mt chromosomes and found that these predicted ends coincide with an abrupt decrease in coverage in our assemblies (Additional file 2). This in combination with the reported sizes of one (*H. magnipapillata *[18]) or both (*H. magnipapillata *and *H. vulgaris *[13]) mt chromosomes from the same or a closely related *Hydra *species suggests that the excess sequences are artifacts, and consequently they were omitted.

To exclude the possibility that other observations in our assemblies originated from methodological artifacts, we conducted additional experimental procedures and tests as follows:

### 1. PCR experiments

PCR experiments were done with a closely related *Hydra *species. We obtained specimens of *Hydra *sp. from the Schulbiologie-Zentrum Hannover. DNA from one polyp was prepared with the Chelex method (protocol as described in [22]), 1 μl of the undiluted supernatant or 1 μl of a 1:10 dilution was used as template. Phylogenetic analysis with partial *cox1 *data verified that our *Hydra *sp. specimen is very closely related to *Hydra magnipapillata *(see Results).

Primers (Additional file 3) were designed to confirm our bioinformatically derived observations via PCR. The fragments shown in Fig. 1 were amplified and sequenced (some in two overlapping parts, see Additional file 3 for details). Sequences have been submitted to GenBank [GenBank: EU683621–EU683624].

### 2. Additional local genome assembly experiments

To exclude the possibility that we had amplified a nuclear mt pseudogene (NUMT) of the *nd5 *and partial *cox1 *fragment, we started different assemblies with the pipeline originating from blast hits of a 200 bp fragment (100 bp both down- and up-stream of the connection of *nd5 *and *cox1 *in the assembly), as well as two assemblies starting from the last 100 bp 3' of *nd5*, and the first 100 bp of the incomplete copy of *cox1*. In the first two cases, only one assembly was received, each time consistent with our former assemblies. By starting with 100 bp of the partial *cox1*, we recovered different assemblies, which were compatible with one chromosomal assembly or the other. In no case did we observe any inconsistencies or any presence of nuclear genes, which would have indicated that the *nd5*-*cox1 *arrangement is part of a NUMT.

#### Phylogenetic analyses

The *H. magnipapillata *and *Hydra*. sp *cox1 *sequences from our assembly and sequencing were manually aligned with additional sequences from other *Hydra *and outgroup species available from GenBank in the SeaView editor [23]. The final dataset contained 560 characters. A maximum likelihood analysis was carried out in PAUP* 4.0b10 [24] under a model of nucleotide evolution suggested by the hierarchical likelihood ratio test in Modeltest 3.7 [25]; for the bootstrap analysis (1,000 replicates) we applied the same model. The dataset was also analyzed with MrBayes v3.1.2 [26,27] (six substitution rates with a proportion of invariant sites, two runs with eight chains each for 2 million generations with a sample frequency of 100 generations, and a burn-in of 50,000 generations). Parameter stabilization of the chains in MrBayes was monitored with Tracer 1.4 [28], and convergence of chains was examined with the diagnostics provided by the AWTY server [29,30].

#### Base compositions of cnidarian mt genomes

Sequences of 23 additional cnidarian genomes were downloaded from GenBank [20] (see Additional file 4 for taxa and accession numbers). The sequences of the 13 respiratory chain protein genes shared between all 24 mt genomes and the sequences of *rns *and *rnl *were extracted from the GenBank format using the Artemis software v.9 [31]. In some mt genomes the rRNA genes were not entirely annotated in their full length. We therefore considered the non-coding regions around the apparently too small genes as rRNAs. The corresponding taxa and positions in each sequence were: *Nematostella *sp. [GenBank: NC_008164] (*rns*: 5054..6171; *rnl*: 10342..12484); *Mussa angulosa *[GenBank: NC_008163] (*rns*: 6901..8038; *rnl*: 15327..17170);* Astrangia *sp. [GenBank: NC_008161] (*rns*: 6899..7797; *rnl*: 12982..14681). The AT contents of rRNAs and protein codon positions of the mt genomes are shown in Additional file 4.

## Results

### Genes, base composition and codon usage

The two chromosomes of the *H. magnipapillata *mt genome each carry one rRNA gene (mt1:*rnl*; mt2: *rns*). Each of the assembled contigs of mt1 and mt 2 is represented by > 7,000 single sequence reads from the trace archive (Additional file 1). Our consensus sequence for mt1 is 8,194 bp long. This matches the length reported by Bridge et al. [14] for this *H. magnipapillata *mt chromosome. The sequence of mt2 is shorter (7,686 bp).

The *H. magnipapillata *mt genome includes 13 protein-coding genes of the respiratory chain usually found in other Metazoa. mt1 contains 6 protein-coding genes, *rnl *and two tRNA genes; mt2 contains 7 protein-coding genes, *rns *and one tRNA gene (Fig. 1A). All genes are unidirectionally encoded on each of the two molecules and densely arranged along the chromosomes. As in *H. oligactis*, the longest non-coding intergenic region is 52 bp between *cox3 *and *nd2 *[17]. Otherwise, subsequent genes are separated by 0–5 bp or overlap for up to 10 bp (in *nd6*-*nd3 *and *nd1*-*nd4*).

Like many other Cnidaria [16,17,32-34], the *H. magnipapillata *mt genome possesses only the two tRNA genes for methionine (*trnM*; CAU) and tryptophan (*trnW*; UCA). *trnW *is only found on mt1, whereas identical copies of *trnM *are present on both chromosomes (Fig. 1B).

**Figure 2:**
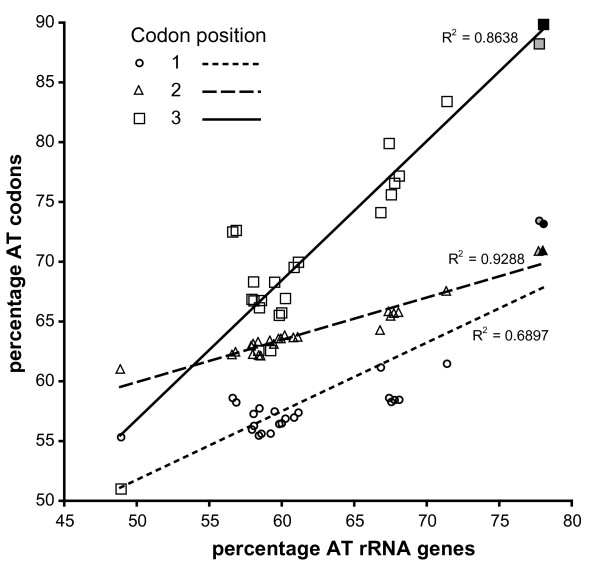
**Base composition in cnidarian mt genomes**. Correlations of AT content (%) of mt rRNAs and the AT content (%) in the codon positions 1, 2 and 3 calculated from 13 protein-coding genes of 24 cnidarian mt genomes (Additional file 4). Black filled symbols = *H. magnipapillata; *grey filled symbols = *H. oligactis*.

Six amino acid codons are not used in the 13 protein-coding genes (Table 1), and all genes are terminated by TAA. Apparently synonymous codons that posses an A or T, instead of a G or C, at the third codon position are preferred in *H. magnipapillata*. To test whether this observation is caused by mechanisms that affect base composition in the whole mt genome, we analyzed codon usage in the 13 respiratory protein-coding genes in 24 mt genomes of Cnidaria. We plotted the AT content at each of the three codon positions against the AT contents of the rRNA genes for every genome, as rRNA coding genes represent a different part of the mt genomes in terms of functional constraints compared to protein-coding genes. Remarkably, *H. magnipapillata *showed the highest values for AT content at the third codon positions (89.8%) and in the rRNA genes (78.1%; Fig. 2, black filled symbols). Moreover, a high AT content in rRNA genes generally correlates with the usage of A and T at third codon positions in all Cnidaria (significant at p = 0.001), suggesting that codon usage might be the result of a general selection for base composition on the mt genome caused by interaction of mutational, repair, replication and translational mechanisms [35]. The AT content at the first and second codon positions also correlates with that of the rRNA genes (significant at p = 0.001), but here AT content rise at a lower rate than the increasing AT content of the rRNAs (regression line slopes: first codon position: 0.46; second: 0.33; third: 1.18). This is likely the result of selection on certain amino acids. Cnidarians posses a lower AT content at the first codon position than at the second (Fig. 2), with *H. magnipapillata *and *H. oligactis *being the only exceptions (73.1% vs. 70.9% for *H. magnipapillata*, filled symbols in Fig. 2).

**Table 1:** Codon usage.

	**Codon**	**n**		**Codon**	**n**		**Codon**	**n**		**Codon**	**n**
**Phe**	TTT	406	**Ser**	TCT	150	**Tyr**	TAT	169	**Cys**	TGT	39
	TTC	32		TCC	15		TAC	14		TGC	2
**Leu**	TTA	456		TCA	95	**TER**	TAA	13	**Trp**	TGA	74
	TTG	33		TCG	2		TAG	**0**		TGG	3
**Leu**	CTT	48	**Pro**	CCT	61	**His**	CAT	72	**Arg**	CGT	6
	CTC	4		CCC	9		CAC	8		CGC	**0**
	CTA	55		CCA	53	**Gln**	CAA	56		CGA	**0**
	CTG	3		CCG	2		CAG	3		CGG	**0**
**Ile**	ATT	304	**Thr**	ACT	92	**Asn**	AAT	217	**Ser**	AGT	79
	ATC	36		ACC	11		AAC	43		AGC	16
	ATA	298		ACA	51	**Lys**	AAA	119	**Arg**	AGA	51
**Met**	ATG	86		ACG	**0**		AAG	11		AGG	**0**
**Val**	GTT	77	**Ala**	GCT	90	**Asp**	GAT	67	**Gly**	GGT	65
	GTC	7		GCC	7		GAC	14		GGC	9
	GTA	84		GCA	42	**Glu**	GAA	82		GGA	111
	GTG	6		GCG	**0**		GAG	3		GGG	26

Codon frequency among the 3,987 codons of the 13 protein-coding genes in *H. magnipapillata*.

### Gene arrangement and inverted terminal repeats

Compared to the gene arrangement of *A. aurita *and *H. oligactis*, only a few changes can be observed in *H. magnipapillata*. Neglecting the positions of tRNAs, two blocks (*cox2*, *atp8*, *atp6*, *cox3*, *nd2*, *nd5 *and *rns*; *nd6*, *nd3*, *nd4L*, *nd1*, *nd4*, *cob*) of genes are identical across the three genomes, occurring on mt1 or mt2, respectively, in *H. magnipapillata *(Fig. 1A). The mt genomes of *H. oligactis *and of *H. magnipapillata *are entirely alignable and display a sequence divergence of 12.3% (excluding the terminal chromosome structures; see below).

As mentioned before, we found 191–196 bp of ITR at both ends of mt1 and mt2. In the linear mt genomes of *H. oligactis *and *A. aurita*, ITR were also present but were longer (*H. oligactis*: 1,488 bp; *A. aurita*: 471 bp, [16,17] assuming symmetry for unsequenced ends). Unlike ITR in *Aurelia *[16], ITR in *H. magnipapillata *have a higher GC content than the rest of the molecule (52.2% GC in ITR vs. 25.2% GC in 5' IOS [see below], 27.6% GC in 3' IOS [see below] and a mean of 22.5% GC for all remaining regions). We found that a smaller part of 3' *cox1 *(54 bp) is included in all ITR of *H. magnipapillata*. Probably because the 3' end of *cox1 *is not very conserved, Pont-Kingdon et al. [18] missed this feature in their mt1 fragment of *H. vulgaris*. The ITR regions of *H. oligactis *contain a larger *cox1 *fragment (one non-functional copy at the 5' end, functional *cox1 *at 3' end, Fig. 1A). The remaining sequenced 3' region of ITR in *H. oligactis *is very similar to those found in *H. magnipapillata *and *H. vulgaris *(Fig. 1C), but longer. Between *H. magnipapillata *and *H. vulgaris*, the major difference is that a stretch of Gs (31 in *H. vulgaris*) is significantly shorter in *H. magnipapillata *(11–16 at the homologous region).

In *H. magnipapillata *mt1 and mt2, we found additional identical sequences at the 5' and 3' ends following (at the 5' ends) and preceding (at the 3' ends) the ITR. We refer to those regions as identically oriented sequences (5' and 3' IOS, Fig. 1B). After the ITR, the 5' IOS of both molecules contain identical copies of non-coding DNA and *trnM*. At the 3' IOS we found a larger partial copy of the 5' region of *cox1 *on mt1. As a consequence of this arrangement, mt1 and mt2 share 310 bp (ITR+5' IOS) at the 5' end and 436 bp (3' IOS+ITR) at the 3' end, giving both molecules a specific orientation.

Using PCR experiments with the closely related *Hydra *sp., we verified the following arrangements initially observed in the *H. magnipapillata *sequences (compare Fig. 1A): (i) the presence and orientation of the ITR at all four chromosome ends could be shown, as well as the presence of partial *cox1 *sequences in the ITR; (ii) identical regions are shared at the 5' and 3' end, respectively, between mt1 and mt2 adjacent to the ITR; and (iii) within the latter regions, the 5' motif contains *trnM*, which therefore appears in two copies in the genome, and a larger sequence of *cox1 *forms the shared 3' motif of mt1 and mt2.

### Phylogenetic analysis

**Figure 3:**
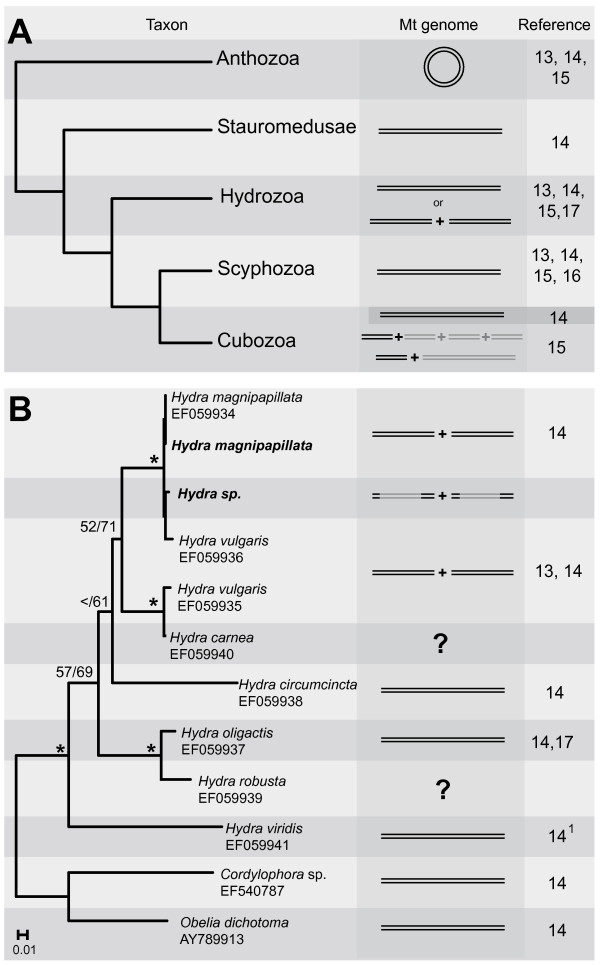
**Evolution of cnidarian mt organization**. **A**. Summary of relationships of higher cnidarian taxa according to nc Small and Large Subunit rRNA data [37], and the organization of mt genomes. Note that in [15] only the size of mt chromosome carrying *rnl *was examined. **B**. Summary of relationships within the genus *Hydra *based upon our ML and Bayesian analyses of partial *cox1 *data, rooted with other hydrozoan sequences from GenBank (accession numbers are given after each species name). Support values >50 are shown above branches (ML bootstraps/Bayesian posterior probability, * = 100 in both analyses). Sequences from this study are bold. Expected mt genome organization is shown in grey. ^1^syn.: *Hydra viridis*; *H. viridissima*.

The tree topology derived from our phylogenetic analysis of *cox1 *shows the close relationship of *Hydra *sp. and *H. magnipapillata *(Fig. 3), thus ensuring that we used an appropriate taxon to test our results. *H. vulgaris *(Two sequences from GenBank) is paraphyletic, which reflects the difficult taxonomy of the genus [36]. The presented phylogeny, in combination with the mt genome organization, supports the view that the ancestral state of mt genome organization in the genus *Hydra *was a single linear mt chromosome.

## Discussion

### Linear mt genomes and fragmentation of mt chromosomes in Cnidaria

Linearity of mt genomes seems to have evolved once after the divergence of Medusozoa from Anthozoa. Fig. 3A summarizes the results of different studies [13,15-17], mapped on a cnidarian phylogeny [37]. A fragmentation of mt genomes has been reported from several *Hydra *species (Hydrozoa) [13,14] and Cubozoa [15]. Uncertainties remain for Cubozoa: Bridge et al. [14] studied the same cubozoan species *Carybdea marsupialis *as Ender and Schierwater [15], but reported a single ~16 kb linear mt genome, while in the more recent work, a ~4 kb fragment was shown to carry the *rnl *gene. Because Ender and Schierwater [15] were able to repeat the experiments with different DNA isolates of *C. marsupialis *and obtained concordant results from an additional cubozoan species (*Tripedalia cystophora*), an experimental error seems unlikely. However, their conclusion of four equally-sized mt chromosomes in Cubozoa is not directly supported by their identification of a 4 kb chromosome carrying *rnl*. Alternatively, one could assume the presence of a single ~12 kb mt counterpart, as indicated in Fig. 3A. Such an arrangement is possible, e.g., if *rnl *and *cox1*, the two genes that are encoded in different orientation to the other mt genes in *A. aurita *[16], were encoded in one chromosome in Cubozoa, and the remaining genes on a second chromosome.

However, given the available data it seems reasonable to assume that fragmented linear genomes occur in both Cubozoa and Hydrozoa (in some members of the genus *Hydra*). This suggests from an evolutionary perspective that the mt genome in the common ancestor of Medusozoa was linear and then independently split into different chromosomes in *Hydra *(Fig. 3B), and in at least some Cubozoa (compare Fig. 3A).

A possible mechanism for the origin of linear chromosomes from a circular molecule is the integration of one or more resolution elements [4]. The circular DNA molecule would be split into one or more linear molecules with identical ends. In Medusozoa, the processes of linearization and the split of one linear into two linear chromosomes were obviously different processes as shown in the phylogenetic trees (Fig. 3). The linearization, possibly occurring in the last common ancestor of medusozoans, seems to have preceded the splitting of the chromosomes by a long time. If the ancestral linear mt chromosome of Medusozoa originated by introduction of a resolution element, one probably would not expect to observe its original motifs, which would occur as identical repeats at the two ends of the linear molecule [4]. Indeed, the ends of linear medusozoan mt chromosomes have inverted terminal motifs (the ITR), instead of direct repeats. The splitting of ancestral linear mt chromosomes as in *H. magnipapillata *(and possibly Cubozoa) happened much later in evolutionary history, contradicting the view that the two or more linear mt chromosomes in Medusozoa directly originated from one circular DNA molecule.

Fragmented mt genomes are present in various eukaryotic taxa, e.g., in dinoflagellates [38,39], Ichthyosporea [12] and Fungi [40]. In Metazoa, fragmented mt genomes are known from the genera *Globodera *(Nematoda [41-43]), *Dicyema *(Mesozoa [44]) and the rotifer *Brachionus plicatilis *[45], but unlike in *H. magnipapillata*, in these taxa the genomes are encoded on several small circular molecules. The mt chromosomal organization observed in *H. magnipapillata *supports the hypothesis of an ancestral, linear chromosome in *Hydra *(Fig. 3B), as represented by the mt genome of *H. oligactis *[17], which has been split in two between *nd5 *and *rns*.

### Function of ITR and IOS

Warrior [13] already suggested the presence of identical terminal sequences on both chromosomes of *H. vulgaris*. We now show that these ends are arranged as ITR on mt1 and mt2, as in other medusozoans [16,17]. In *H. oligactis*, which in the phylogenetic tree branches off before *Hydra *species carrying two mt DNA molecules (Fig. 3B), the single linear mt chromosome has ITR containing a large copy of the 5' end of *cox1*. Only the ITR at the 3' end has been completely sequenced [17]. Based on our findings in *H. magnipapillata*, we predict that the unsequenced 5' end is almost identical to the 3' motif (Fig. 1), and we expect that about 150 bp remain unsequenced on the 5' end (in contrast to the 65 bp that have been proposed [17]). In *Hydra*, partial copies of *cox1 *play a crucial role as part in ITR regions at the chromosome ends (Fig. 1, [17]). The ITR in *H. magnipapillata *contains only a short sequence of the 3' end of *cox1 *(54 bp, compared to the 1,284 bp in *H. oligactis*), suggesting that large parts of the *cox1 *copies were lost. A simultaneous duplication of 5' ITR (containing the already shortened partial *cox1 *copy) and the 5' IOS motif seems likely to have occurred in the process of chromosome splitting. In this case, the longer *cox1 *copy (containing additional 240 bp of *cox1*) is a duplication of the functional *cox1 *of the original 5' end of a single mt chromosome (Fig. 1A).

ITR of linear mt molecules are present in other taxa besides medusozoans, e.g., in yeasts (e.g., [9]) and in the green algae *Chlamydomonas reinhardtii *[10]. Furthermore, in the green algae *Polytomella parva*, identical ITR are present at all ends of the two linear mt chromosomes [11], similar to what we observe in *H. magnipapillata*. We report 5' and 3' IOS as an additional shared feature of the two mt chromosomes. Interestingly, such an arrangement of ITR and 5' and 3' IOS is also seen in another, highly fragmented eukaryotic mt genome. In the ichthyosporean *Amoebidium parasiticum*, mt genes are distributed over several hundred different chromosomes, each of which also possesses ITR and 5' and 3' IOS [12].

Pont-Kingdon et al. [18] speculated that there may be a role for transcription initiation at the 240 bp 5' of *trnM*, which they found in their *H. vulgaris *(as *H. attenuata*) partial mt1 sequence. Considering that transcription initiation within the ITR would result in energetically expensive nonsense transcripts (since all genes are encoded on only one strand), transcription is more likely to start in the adjacent, non-coding regions of the 5' IOS. In *H. magnipapillata *and *H. vulgaris *this region within the 5' IOS is 40 bp long and lies between the *cox1 *copy and *trnM *(Fig. 1B). In *H. oligactis*, the non-coding region between the ITR and *trnM *is only 6 bp. However, a striking sequence similarity can be observed near *trnM *between *H. oligactis *and *H. vulgaris *(with the same sequence in this region as *H. magnipapillata*) [17]. There is a 14-bp motif (TTATTTRRTCTTCT) that is shared between the species and differs by the last 3 bp from the 3' ITR+3 bp counterpart in *H. oligactis*. This motif might be involved in transcription initiation. If so, the difference in the very last 3 bp between the 5' end and its counterpart on the reverse strand in the ITR of *H. oligactis *prevents a functional transcription signal on the non-coding strand in this species. A crucial function for transcription initiation would explain selective pressure for maintaining the 5' IOS of both molecules after the ITR in *H. magnipapillata*. All mt chromosomes from *Amoebidium parasiticum *that contain coding genes are transcribed from 5' IOS to 3' IOS, as in *H. magnipapillata *[12]. This observation led Burger et al. [12] to the conclusion that the IOS in *Amoebidium *are responsible for transcription initiation (5' IOS) and termination (3' IOS). While in *H. magnipapillata *we expect the same function for 5' IOS, the role of the additional partial *cox1 *copy within the 3' IOS of mt1 and mt2, if any, remains unknown; considering that the end of *cox1 *is part of the ITR, transcription can only be terminated in ITR and not in the 3' IOS. The sequence homologies of ITR and IOS within or between mt1 and mt2 are probably not the result of a relatively recent origin from ancestral sequences, as a first duplication of partial *cox1 *is already observed in *H. oligactis *and therefore predates the separation process. The substitutions between ITR (partial *cox1*) of the two species are found in all ITR copies in each mt genome. Considering this and the fact that similar arrangements are found in other eukaryotes [8-12,16], it seems more likely that concerted evolution maintains the almost identical sequences, probably caused or influenced by the yet unknown mt genome replication mechanism. Terminal sequences of linear DNA molecules play a crucial role in mt replication [4]. The main problem in the process of linear chromosome replication is the maintenance of the 5' ends. In nuclear (nc) chromosomes this is normally achieved by telomerase, an enzyme that adds short sequence motifs in tandem repeats at the end of each molecule to compensate for loss at the 5' end that occurs in each replication cycle [46]. Consequently, in *Hydra *as in most Metazoa, the motif (TTAGGG)_n _is found at the end of nc chromosomes [47]. The termini of *Hydra *mt chromosomes are much more complex, and their maintenance during replication is not yet understood but is most likely telomerase independent [13]. Warrior [13] suggested an mt replication mechanism for the two *H. vulgaris *mt chromosomes similar to that of T7 bacteriophages. His conclusion was based upon observations from hybridization experiments, which showed the presence of identical terminal sequences that he assumed to have the same orientation at the 5' and 3' ends. According to this model, intermediate concatamers are formed, and via ligation, site-specific nicking and elongation, the 5' ends are finally filled. Based on our data we can reject this model, because we showed that the terminal sequences in *H. magnipapillata *are inverted (ITR) and therefore do not allow the necessary concatamerization in the proposed way. Similarly, ephemeral circularization as in the phage lambda is not possible when terminal sequences are not direct repeats, but inverted.

Different replication mechanisms for linear chromosomes have been reported or proposed (for review, see [48]). Solutions for maintaining the terminal structure in sequences with ITR include (a) covalently bound proteins, which also could serve as primers for a 'racket frame' replication (e.g., in linear mt chromosomes of plants and fungi or adenoviruses [48,49]), (b) 5' and 3' ends of chromosomes that are connected by a hairpin-loop (e.g., in some yeasts [9,48]) and (c) single-stranded 3' overhangs (e.g., in *Chlamydomonas reinhardtii *[10]). Warrior [13] showed that the first two possibilities are not realized in *H. vulgaris*. The mt-replication mechanism in *Chlamydomonas *requires an internal repeat of the single-stranded 3' overhangs [10]. We cannot entirely rule out the existence of short single-stranded 3' overhangs, as it is possible they might have been missed due to our methods. The outermost sequence at least cannot be part of a repeat motif, as our PCR amplification of *Hydra *sp. did not yield fragments of different sizes. Furthermore, neither in mt1 of *H. vulgaris *nor in the mt genome of *H. oligactis *were additional sequences or repeats found [13,17]. Therefore, although a similarity to the mt replication of *C. reinhardtii *cannot be excluded, we find that mechanisms for linear mt chromosome replication are too diverse and that too many details are still unknown [4,48] for us to draw further conclusions about the replication process in *H. magnipapillata *from the presence of the ITR alone. Considering that ITR are a shared feature among all three available sequences of medusozoan mt genomes (*H. oligactis *[16,17], *A. aurita *[16,17] and *H. magnipapillata*), it is very likely that the mechanisms for mt replication are similar in all medusozoans and are still the same after the fragmentation of the mt genome. Keeping in mind that similar arrangements of ITR and IOS are found in *Amoebidium parasiticum*, the mt replication mechanisms of *H. magnipapillata *and Medusozoa are probably not unique among eukaryotes.

## Conclusion

The *H. magnipapillata *mt genome represents the first complete sequence of a linear metazoan mt genome that consists of two separate molecules. The gene arrangements and our phylogenetic analysis suggest that mt1 and mt2 originated from an ancestral linear mt genome, as found in *H. oligactis*, that at some point divided in two between *nd5 *and *rns *(Figs. 1 and 3). Of most interest is the organization at the ends of the two mt chromosomes (Fig. 1). We show that *H. magnipapillata *has ITR, which include a part of *cox1 *(at the 3' ITR of mt2) or partial copies of the 5' end of *cox1 *(all other ITR). We conclude that mechanisms for mt replication in *Hydra *species are different from the previously proposed ones and probably are shared among all medusozoans. In addition to ITR, both mt chromosomes of *H. magnipapillata *have identical motifs on their 5' and 3' ends, called respectively the 5' IOS and 3' IOS (Fig. 1). The 5' IOS includes *trnM *and a non-coding region, including a motif that may play a role in transcription initiation. The 5' IOS is probably the result of a duplication during the separation process of a single ancestral mt chromosome. The organization of the ITR and 5' and 3' IOS is not unique among eukaryotes with fragmented linear mt genomes. ITR most likely play a role in mt replication, while the duplication of the 5' end of an single ancestral linear and unidirectionally-encoded mt chromosome (with the presence of 5' IOS) and its concerted evolution ensure that transcription of all mt genes is maintained after fragmentation of linear mt chromosomes. A similar arrangement of ITR and IOS regions can therefore be expected in the apparently fragmented mt genomes of Cubozoa and other eukaryotes with two or more linear mt DNA molecules.

## Authors' contributions

OV contributed to the conception and design of the study, acquired, analyzed and interpreted the data, and wrote the manuscript. DE and GW contributed to the interpretation of data and critically reviewed the manuscript. All authors read and approved the final version of the manuscript.

## Supplementary Material

Additional file 1 Trace IDs of sequences included in the *H. magnipapillata *assemblies. Click here for file

Additional file 2 Coverage of mt1 and mt2 assemblies. Click here for file

Additional file 3 Primer sequences. Click here for file

Additional file 4 Taxa, GenBank accession numbers and AT contents of protein-coding genes and rRNA genes in 24 cnidarian mt genomes. Click here for file

## References

[B1] Gray MW, Burger G, Lang BF (1999). Mitochondrial evolution. Science.

[B2] Lang BF, Gray MW, Burger G (1999). Mitochondrial genome evolution and the origin of eukaryotes. Annu Rev Genet.

[B3] Burger G, Gray MW, Lang BF (2003). Mitochondrial genomes: anything goes. Trends Genet.

[B4] Nosek J, Tomaska L (2003). Mitochondrial genome diversity: evolution of the molecular architecture and replication strategy. Curr Genet.

[B5] Gray MW, Lang BF, Burger G (2004). Mitochondria of protists. Annu Rev Genet.

[B6] Boore JL (1999). Animal mitochondrial genomes. Nucleic Acids Res.

[B7] Lavrov DV (2007). Key transitions in animal evolution: a mitochondrial DNA perspective. Integr Comp Biol.

[B8] Pritchard AE, Cummings DJ (1981). Replication of linear mitochondrial DNA from *Paramecium*: sequence and structure of the initiation-end crosslink. Proc Natl Acad Sci USA.

[B9] Dinouel N, Drissi R, Miyakawa I, Sor F, Rousset S, Fukuhara H (1993). Linear mitochondrial DNAs of yeasts: closed-loop structure of the termini and possible linear-circular conversion mechanisms. Mol Cell Biol.

[B10] Vahrenholz C, Riemen G, Pratje E, Dujon B, Michaelis G (1993). Mitochondrial DNA of *Chlamydomonas reinhardtii*: the structure of the ends of the linear 15.8-kb genome suggests mechanisms for DNA replication. Curr Genet.

[B11] Fan J, Lee RW (2002). Mitochondrial genome of the colorless green alga *Polytomella parva*: Two linear DNA molecules with homologous inverted repeat termini. Mol Biol Evol.

[B12] Burger G, Forget L, Zhu Y, Gray MW, Lang BF (2003). Unique mitochondrial genome architecture in unicellular relatives of animals. Proc Natl Acad Sci USA.

[B13] Warrior R (1987). The mitochondrial DNA of *Hydra attenuata *consists of two linear molecules.

[B14] Bridge D, Cunningham CW, Schierwater B, DeSalle R, Buss LW (1992). Class-level relationships in the phylum Cnidaria: evidence from mitochondrial genome structure. Proc Natl Acad Sci USA.

[B15] Ender A, Schierwater B (2003). Placozoa are not derived cnidarians: evidence from molecular morphology. Mol Biol Evol.

[B16] Shao Z, Graf S, Chaga OY, Lavrov DV (2006). Mitochondrial genome of the moon jelly *Aurelia aurita *(Cnidaria, Scyphozoa): A linear DNA molecule encoding a putative DNA-dependent DNA polymerase. Gene.

[B17] Kayal E, Lavrov DV (2008). The mitochondrial genome of *Hydra oligactis *(Cnidaria, Hydrozoa) sheds new light on animal mtDNA evolution and cnidarian phylogeny. Gene.

[B18] Pont-Kingdon G, Vassort CG, Warrior R, Okimoto R, Beagley CT, Wolstenholme DR (2000). Mitochondrial DNA of *Hydra attenuata *(Cnidaria): a sequence that includes an end of one linear molecule and the genes for l-rRNA, tRNA(f-Met), tRNA(Trp), COII, and ATPase8. J Mol Evol.

[B19] Altschul SF, Madden TL, Schaffer AA, Zhang J, Zhang Z, Miller W, Lipman DJ (1997). Gapped BLAST and PSI-BLAST: a new generation of protein database search programs. Nucleic Acids Res.

[B20] National Center for Biotechnology Information (GenBank). http://www.ncbi.nlm.nih.gov/.

[B21] Huang X, Madan A (1999). CAP3: A DNA sequence assembly program. Genome Res.

[B22] Voigt O, Collins AG, Pearse VB, Pearse JS, Ender A, Hadrys H, Schierwater B (2004). Placozoa – no longer a phylum of one. Curr Biol.

[B23] Galtier N, Gouy M, Gautier C (1996). SEAVIEW and PHYLO_WIN: two graphic tools for sequence alignment and molecular phylogeny. Comput Appl Biosci.

[B24] Swofford DL (2003).

[B25] Posada D, Crandall KA (1998). MODELTEST: testing the model of DNA substitution. Bioinformatics.

[B26] Ronquist F, Huelsenbeck JP (2003). MrBayes 3: Bayesian phylogenetic inference under mixed models. Bioinformatics.

[B27] Huelsenbeck JP, Ronquist F (2001). MRBAYES: Bayesian inference of phylogenetic trees. Bioinformatics.

[B28] Tracer v1.4. http://tree.bio.ed.ac.uk/software/tracer/.

[B29] AWTY web application. http://king2.scs.fsu.edu/CEBProjects/awty/awty_start.php.

[B30] Nylander JA, Wilgenbusch JC, Warren DL, Swofford DL (2008). AWTY (Are We There Yet?): a system for graphical exploration of MCMC convergence in Bayesian phylogenetics. Bioinformatics.

[B31] Rutherford K, Parkhill J, Crook J, Horsnell T, Rice P, Rajandream MA, Barrell B (2000). Artemis: sequence visualization and annotation. Bioinformatics.

[B32] Beagley CT, Okimoto R, Wolstenholme DR (1998). The mitochondrial genome of the sea anemone *Metridium senile *(Cnidaria): Introns, a paucity of tRNA genes, and a near-standard genetic code. Genetics.

[B33] Brugler MR, France SC (2007). The complete mitochondrial genome of the black coral *Chrysopathes formosa *(Cnidaria:Anthozoa:Antipatharia) supports classification of antipatharians within the subclass Hexacorallia. Mol Phylogenet Evol.

[B34] Medina M, Collins AG, Takaoka TL, Kuehl JV, Boore JL (2006). Naked corals: skeleton loss in Scleractinia. Proc Natl Acad Sci USA.

[B35] Perna NT, Kocher TD (1995). Patterns of nucleotide composition at fourfold degenerate sites of animal mitochondrial genomes. J Mol Evol.

[B36] Hemmrich G, Anokhin B, Zacharias H, Bosch TC (2007). Molecular phylogenetics in *Hydra*, a classical model in evolutionary developmental biology. Mol Phylogenet Evol.

[B37] Collins AG, Schuchert P, Marques AC, Jankowski T, Medina M, Schierwater B (2006). Medusozoan phylogeny and character evolution clarified by new large and small subunit rDNA data and an assessment of the utility of phylogenetic mixture models. Syst Biol.

[B38] Slamovits CH, Saldarriaga JF, Larocque A, Keeling PJ (2007). The highly reduced and fragmented mitochondrial genome of the early-branching dinoflagellate *Oxyrrhis marina *shares characteristics with both apicomplexan and dinoflagellate mitochondrial genomes. J Mol Biol.

[B39] Jackson CJ, Norman JE, Schnare MN, Gray MW, Keeling PJ, Waller RF (2007). Broad genomic and transcriptional analysis reveals a highly derived genome in dinoflagellate mitochondria. BMC Biol.

[B40] Burger G, Lang BF (2003). Parallels in genome evolution in mitochondria and bacterial symbionts. IUBMB Life.

[B41] Armstrong MR, Blok VC, Phillips MS (2000). A multipartite mitochondrial genome in the potato cyst nematode *Globodera pallida*. Genetics.

[B42] Gibson T, Blok VC, Phillips MS, Hong G, Kumarasinghe D, Riley IT, Dowton M (2007). The mitochondrial subgenomes of the nematode *Globodera pallida *are mosaics: evidence of recombination in an animal mitochondrial genome. J Mol Evol.

[B43] Gibson T, Blok VC, Dowton M (2007). Sequence and characterization of six mitochondrial subgenomes from *Globodera rostochiensis*: multipartite structure is conserved among close nematode relatives. J Mol Evol.

[B44] Watanabe KI, Bessho Y, Kawasaki M, Hori H (1999). Mitochondrial genes are found on minicircle DNA molecules in the mesozoan animal *Dicyema*. J Mol Biol.

[B45] Suga K, Mark Welch DB, Tanaka Y, Sakakura Y, Hagiwara A (2008). Two circular chromosomes of unequal copy number make up the mitochondrial genome of the rotifer *Brachionus plicatilis*. Mol Biol Evol.

[B46] Nosek J, Kosa P, Tomaska L (2006). On the origin of telomeres: a glimpse at the pre-telomerase world. BioEssays.

[B47] Traut W, Szczepanowski M, Vítková M, Opitz C, Marec F, Zrzavý J (2007). The telomere repeat motif of basal Metazoa. Chromosome Res.

[B48] Nosek J, Tomaska LU, Fukuhara H, Suyama Y, Kovac L (1998). Linear mitochondrial genomes: 30 years down the line. Trends Genet.

[B49] Sakaguchi K (1990). Invertrons, a class of structurally and functionally related genetic elements that includes linear DNA plasmids, transposable elements, and genomes of adeno-type viruses. Microbiol Rev.

