# Supplementary material for: A fragmented metazoan organellar genome: the two mitochondrial chromosomes of Hydra magnipapillata
Source: BMC Genomics. 2008 Jul 26;9:350. doi: 10.1186/1471-2164-9-350 (PMC2518934; doi:10.1186/1471-2164-9-350)
Supplement: Additional file 2 — Coverage of mt1 and mt2 assemblies. [file 1471-2164-9-350-S2.pdf]

Coverage of assemblies for mt1 and mt 2

The lines mark the assumed 5’ (green lines) and 3’ (red lines) end of the molecules. The 5’ end (arrows) of mt1 was inferred from sequence similarity with the experimentally recovered end for *Hydra vulgaris* (as *H. attenuata*) (Warrior 1998). Other ends of mt1 and mt2 were recovered considering former results [13] of identical ends of each molecule (note that in [13] no information about the orientation of the ends was included).

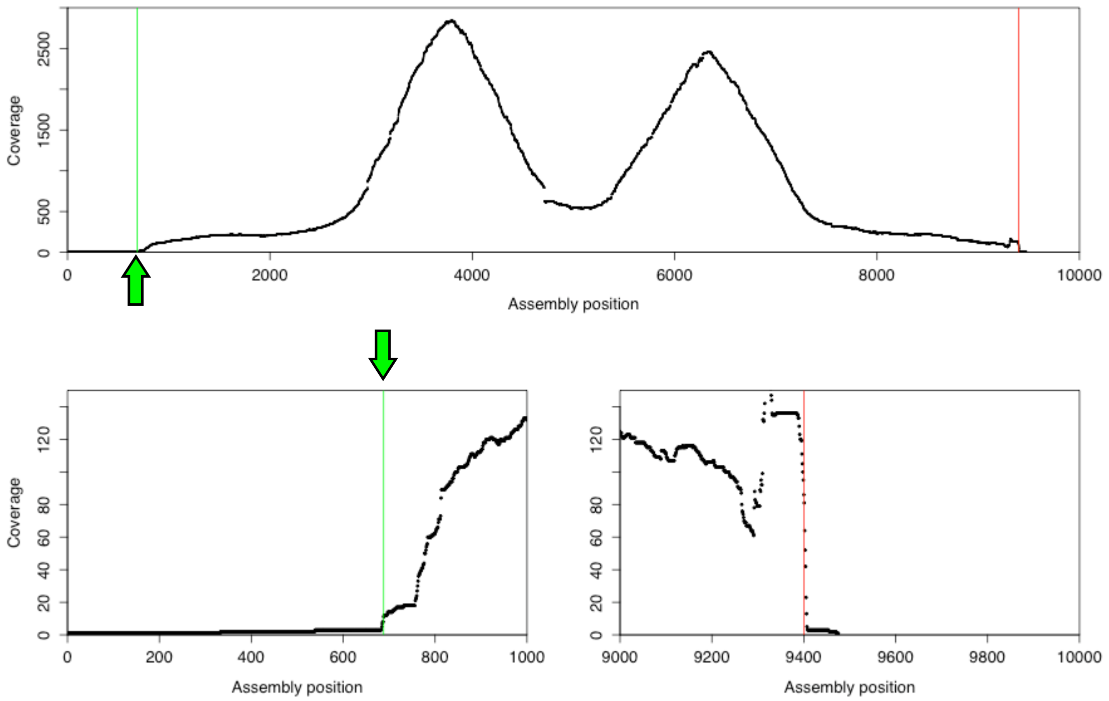

Supplementary fig. 1: Coverage (number of traces) along the mt1 assembly. Assembly positions refer to the consensus sequence including gaps. Top: complete overview, bottom: excerpts from the assembly ends.

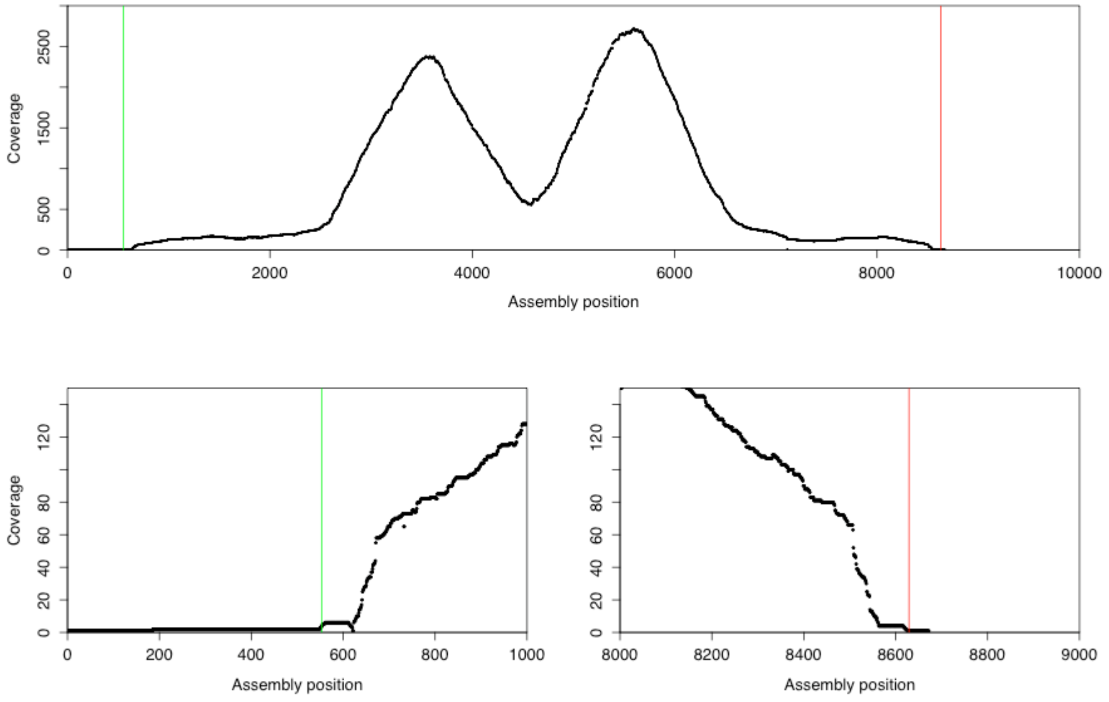

Supplementary fig. 2 Coverage (number of traces) along the mt2 assembly. Assembly positions refer to the consensus sequence including gaps. Top: complete overview, bottom: excerpts from the assembly ends.
