# Supplementary material for: A fragmented metazoan organellar genome: the two mitochondrial chromosomes of Hydra magnipapillata
Source: BMC Genomics. 2008 Jul 26;9:350. doi: 10.1186/1471-2164-9-350 (PMC2518934; doi:10.1186/1471-2164-9-350)
Supplement: Additional file 3 — Primer sequences. [file 1471-2164-9-350-S3.pdf]

Primer sequences used in the PCR experiments [with the following PCR program: 95°C/5min; 37x (95°C/30s; 48°C/30s; 72°C/1-2min; 72°C/3min)] and cycle sequencing reactions. *Primer only used for sequencing. Letters designate two overlapping PCR fragments (a+b).

**Primer name**	**Sequence (5'-3')**	**PCR fragment**	**5' position in *H. magnipapillata***
Hydra_mt_ITR1	CTGCGATAGCCGCAG	2a,3,4b	mt1: 24; 8157<br>mt2: 24; 7648
Hydra_ITR2-seq	GGGTGATCCTCTTTTAGGAG	1, 2*,3*,4b*	mt1: 85; 8091<br>mt2: 81; 7583
Hydra_rnl-rv	CATGAAAAACCAGCTATCTC	1	mt1: 751
Hydra_nd5_1fw	TGATTACCTGATGCDATGG	2b	mt1: 6621
Hydra_nd5_2fw	TTGAAATGTTATCTTTACAACTT	2a	mt1: 7463
Hydra_cox1_rv	CTTCTAGGCATTCCTGCTAA	2b,4a	mt1: 7787<br>mt2: 7279
Hydra_rns-rv	CGTCTGCTGGCACTTA	3	mt2: 562
C1-L1490 (=cox1_fwd) *	GGTCAACAAATCATAAAGATATTGG	4a	mt2: 6005
Hydra_cox1fw2	ACTGTAGGAATGGATGTTGA	4b	mt2: 6862

* Folmer O, Black M, Hoeh W, Lutz R, Vrijenhoek R: **DNA primers for amplification of mitochondrial cytochrome C oxidase subunit I from diverse metazoan invertebrates.** *Molecular Marine Biology and Biotechnology* 1994, **3**:294-299.
